# Lassa Virus Infection of Primary Human Airway Epithelial Cells

**DOI:** 10.3390/v17050592

**Published:** 2025-04-22

**Authors:** Helena Müller-Kräuter, Sarah Katharina Fehling, Lucie Sauerhering, Birthe Ehlert, Janine Koepke, Juliane Schilling, Mikhail Matrosovich, Andrea Maisner, Thomas Strecker

**Affiliations:** 1 Institute of Virology, Philipps University Marburg, Hans-Meerwein-Str. 2, 35043 Marburg, Germany; helena.mueller@staff.uni-marburg.de (H.M.-K.); fehling@staff.uni-marburg.de (S.K.F.); lucie.sauerhering@staff.uni-marburg.de (L.S.); birthe.ehlert@mail.de (B.E.); juliane.schilling@biotest.com (J.S.); matrosov@staff.uni-marburg.de (M.M.); maisner@staff.uni-marburg.de (A.M.); 2 Department of Internal Medicine, Molecular Pneumology, Cardio-Pulmonary Institute, Justus-Liebig University of Giessen, Aulweg 130, 35392 Gießen, Germany; janine.koepke@innere.med.uni-giessen.de

**Keywords:** Lassa mammarenavirus, virus–host interactions, primary airway epithelial cells, IFN-λ

## Abstract

Lassa mammarenavirus (LASV), a member of the family Arenaviridae, is a highly pathogenic virus capable of causing severe systemic infections in humans. The primary host reservoir is the Natal multimammate mouse (*Mastomys natalensis*), with human infections typically occurring through mucosal exposure to virus-containing aerosols from rodent excretions. To better understand the molecular mechanisms underlying LASV replication in the respiratory tract, we utilized differentiated primary human airway epithelial cells (HAECs) grown under air–liquid interface conditions, closely mimicking the bronchial epithelium in vivo. Our findings demonstrate that HAECs are permissive to LASV infection and support productive virus replication. While LASV entry into polarized HAECs occurred through both apical and basolateral surfaces, progeny virus particles were predominantly released from the apical surface, consistent with an intrinsic apical localization of the envelope glycoprotein GP. This suggests that apical virus shedding from infected bronchial epithelia may facilitate LASV transmission via airway secretions. Notably, limited basolateral release at later stages of infection was associated with LASV-induced rearrangement of the actin cytoskeleton, resulting in compromised epithelial barrier integrity. Finally, we demonstrate that LASV-infected HAECs exhibited a pronounced type III interferon response. A detailed understanding of LASV replication and host epithelial responses in the respiratory tract could facilitate the development of targeted future therapeutics.

## 1. Introduction

Lassa mammarenavirus (LASV), an Old World member of the Arenaviridae family, is the causative agent of Lassa fever, an acute systemic febrile disease endemic to several West African countries. Spillover risk models estimate up to 900,000 infections per year [1]. While most infections are mild or asymptomatic, approximately 15–20% of patients hospitalized with severe Lassa fever die from the illness [2,3]. Despite its substantial public health impact, there are currently no approved vaccines or effective treatments for LASV. The only effective drug, the nucleoside analogue ribavirin, is associated with severe side effects and shows efficacy only when administered early in the course of infection [4,5].

LASV is an enveloped virus with a bi-segmented, single-stranded RNA genome that encodes four viral genes using an ambisense coding strategy [6]. The small RNA segment (S-RNA) encodes the nucleoprotein (NP) and the glycoprotein precursor (GPC), while the large RNA segment (L-RNA) encodes the RNA-dependent RNA polymerase L and the small RING finger protein Z [7,8]. The biologically inactive GPC undergoes co- and post-translational cleavage steps that are essential for its maturation and viral infectivity [9,10,11]. The mature glycoprotein (GP) spike on the viral surface is assembled as a trimeric complex consisting of the stable signal peptide (SSP), the receptor-binding subunit GP1, and the transmembrane fusion subunit GP2 [12].

LASV exhibits a broad cell tropism and exploits multiple cellular receptors. The α subunit of dystroglycan (DG) is the primary cell surface receptor [13]. α-DG is widely expressed in many tissues; however, α-DG is subjected to tissue-specific differential glycosylation that may determine LASV host cell tropism [14,15]. The expression of α- and β-DG has been identified in human airway epithelial cells, where it plays a vital role in the migration and repair of these cells following injury [16,17]. Other identified cell surface receptors include DC-SIGN (dendritic cell-specific intercellular adhesion molecule 3-grapping non-integrin), LSECtin (liver and lymph node sinusoidal endothelial calcium-dependent lectin), and the TAM receptor protein tyrosine kinases Axl and Tyro3 [18]. Axl and Tyro3 exhibit widespread distribution in mammalian tissue, performing unique functions in specific cell types [19]. DC-SIGN is expressed on dendritic cells and some subsets of macrophages, while LSECtin is expressed predominantly by sinusoidal endothelial cells of the human liver and lymph nodes [20,21,22]. In addition to these cell surface molecules, LASV requires the intracellular receptor LAMP1 (lysosomal-associated membrane protein 1) for virus entry [23].

LASV is maintained by chronic infection of the Natal multimammate mouse *Mastomys natalensis*, the primary natural host reservoir [24]. Transmission of LASV from rodents to humans is primarily thought to occur via inhalation of dust or droplets contaminated with infectious rodent excretions [25,26,27]. Autopsy and biopsy samples of fatal human Lassa fever cases have demonstrated high viral loads in the liver, spleen, kidneys, and lungs [28]. Additionally, high virus concentrations were detected in the lungs of experimentally infected guinea pigs and mice, where interstitial pneumonia was the most consistent and severe histological lesion [29,30,31]. Collectively, these data indicate that the respiratory tract represents an important replication site of LASV [28].

The respiratory tract is lined with a continuous epithelial cell layer composed of various cell types, depending on the airway section, forming a polarized mechanical barrier against invading pathogens [32,33]. The epithelial barrier prevents viral entry and spread into the submucosa and restricts access to virus-specific cellular receptors on the basolateral membrane [34]. The mechanism by which LASV overcomes the protective barrier of the airway epithelium remains unknown. In previous studies, we demonstrated that LASV entry predominantly occurs via the basolateral membrane in polarized kidney epithelial cells, consistent with the basolateral localization of α-DG in these cells [35].

To better understand the mechanisms underlying LASV infection in the human respiratory tract, we developed an in vitro model of LASV infection using differentiated primary human airway epithelial cells (HAECs). One of the most commonly used models for studying the human respiratory tract is the air–liquid interface (ALI) cell culture, in which human primary epithelial cells are grown with their apical surface exposed to air [36]. ALI cultures closely resemble the composition, structure, and functional properties of the human airway epithelium in vivo [37,38]. Here, we utilized primary HAEC as an in vitro cell culture model to analyze LASV replication kinetics and the polarity of viral entry and release in the bronchial epithelium. We also investigated the induction of type I and III interferon in response to LASV infection. These findings contribute to our understanding of the relevance of the human respiratory tract as an LASV entry and replication site and its potential role in human-to-human transmission via airway secretions.

## 2. Materials and Methods

### 2.1. Viruses and Cell Culture

African green monkey kidney cells (VeroE6, ATCC CRL-1586; American Type Culture Collection, Manassas, VA, USA) and human foreskin fibroblasts (HFFs, ATCC SCRC-1041) were maintained in Dulbecco’s modified Eagle’s medium (DMEM; Gibco, Waltham, MA, USA), supplemented with 10% (*v*/*v*) fetal bovine serum (Invitrogen, Carlsbad, CA, USA), 2 mM L-glutamine (Gibco), 50 U/mL penicillin, and 50 µg/mL streptomycin (Gibco).

For LASV stock production, the LASV strain Josiah was grown in VeroE6 cells and stored at −80 °C. Viral titers were determined by defining the 50% tissue culture infectious dose (TCID_50_) as described previously [39]. All experiments with infectious LASV were performed in the biosafety level (BSL) 4 facilities of the Institute of Virology at Philipps University Marburg, Germany, in compliance with German regulations. Recombinant vesicular stomatitis virus (VSV) expressing LASV strain Josiah-derived GP in place of the original VSV G (VSVΔG/LASVGP) was grown in VeroE6 cells as described previously [40]. VSVΔG/LASVGP was titered by plaque assay using VeroE6 cells with an Avicel RC-591 overlay [41]. Cells were fixed in 10% formaldehyde solution containing 0.1% (*w*/*v*) crystal violet and incubated for 30 min at room temperature (RT). Viral titers are reported as plaque-forming units (PFUs). The influenza A virus (IAV) A/Hamburg/5/2009 (H1N1) was described previously [42].

### 2.2. Air-Liquid Interface Cultures

**Figure 1:**
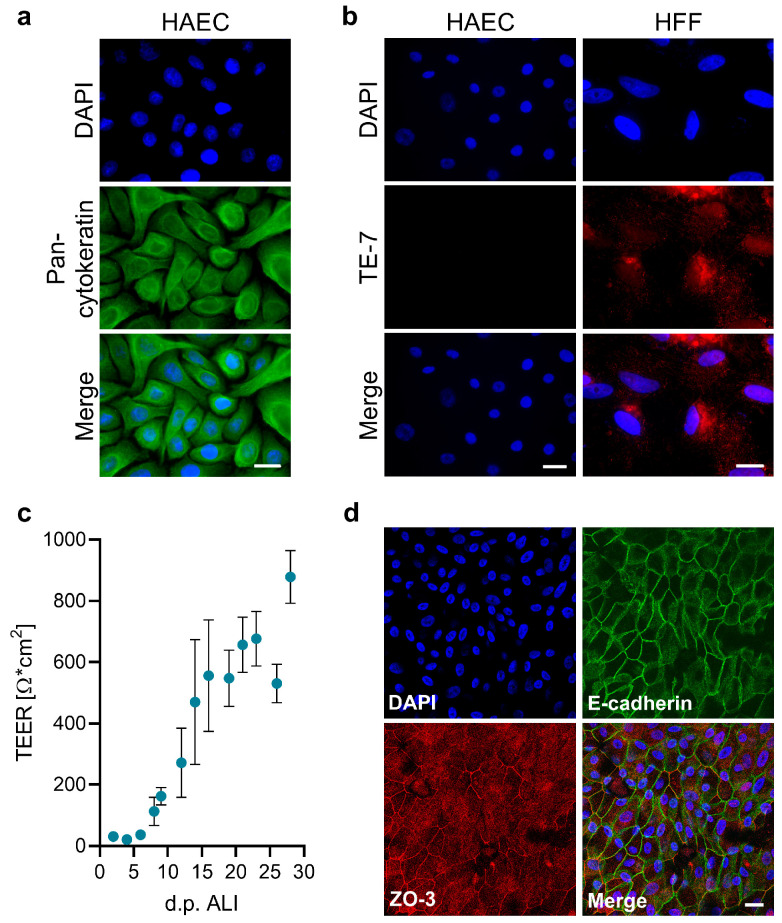
Formation of a polarized phenotype in HAECs. (**a**,**b**) HAECs and HFF cells were seeded on coverslips. After fixation with 4% PFA and permeabilization, cells were immunostained for pan-cytokeratin (**a**) or the fibroblast marker TE-7 (**b**). Nuclei were visualized by DAPI staining. Scale bars, 20 μm. (**c**) HAECs were cultivated on Transwell inserts under ALI culture conditions. TEER was measured after medium changes every 2–3 days following ALI induction. Mean values and standard errors of the mean (SEM) of three independent experiments are shown. (**d**) ALI cultures grown for 35 days were fixed with 4% PFA and permeabilized with Triton X-100, followed by immunostaining of tight and adherens junction proteins (ZO-3, red; E-cadherin, green). Nuclei were visualized by DAPI staining. Confocal images are shown. Scale bars, 20 μm.

**Figure 2:**
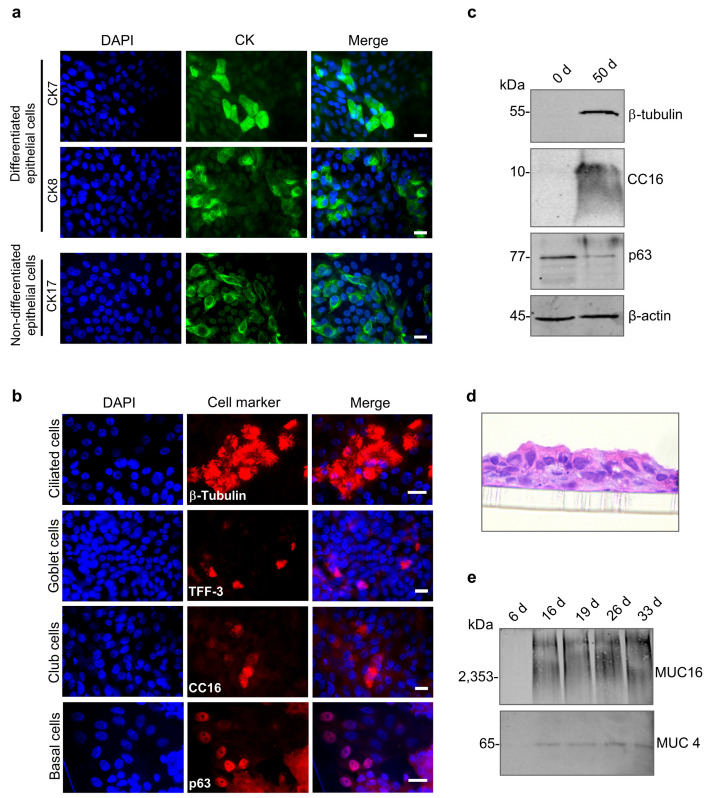
Formation of a differentiated respiratory epithelium. HAECs were seeded on Transwell inserts and cultivated under ALI conditions. (**a**) Immunostaining of specific cytokeratins (CK7, CK8, and CK17). (**b**) Immunostaining of markers for ciliated cells (β-tubulin), goblet cells (TFF-3), club cells (CC16), or basal cells (p63). Cells were analyzed between days 28 and 35 post-airlift. Nuclei were visualized using DAPI staining. Scale bars, 20 μm. (**c**) Western blot detection of ß-tubulin, CC16, and p63 in non-differentiated HAECs (0 d) and HAECs cultured for 50 days under ALI conditions (50 d). (**d**) HAECs were grown on Transwell inserts, followed by histological sectioning and HE staining. Representative histological sections were examined by light microscopy (40× magnification). (**e**) ALI cultures were rinsed weekly, and the apical secretion of mucus proteins (MUC16 and MUC4) was monitored by western blot analysis.

**Figure 3:**
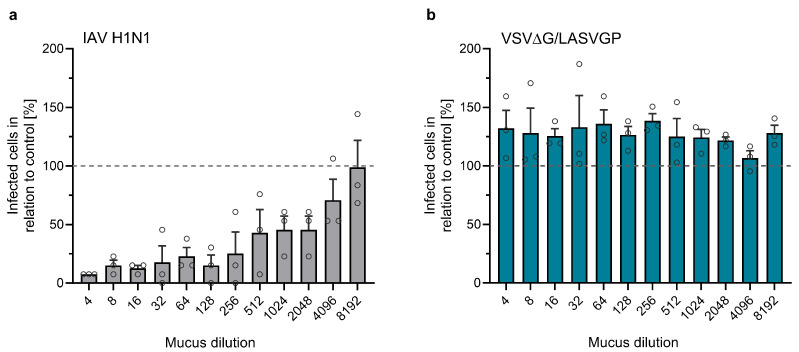
Effect of airway mucus on virus entry. MDCK II (**a**) or VeroE6 (**b**) cells were seeded in a 96-well plate and cultivated for 24 h. Mucus collected from the apical sides of HAECs was diluted and incubated with 1400 FFU of IAV (**a**) or 800 PFU of VSVΔG/LASVGP (**b**). Mixtures of mucus and virus were then added to the cells and incubated for 8 h. Infected cells were detected by immunostaining using anti-IAV NP (**a**) or anti-VSV antibodies (**b**). The total numbers of infected cells per well were quantified. The numbers of infected cells in control samples (virus without mucus) were set as 100% (indicated by dashed lines). Error bars represent SEM of three biological replicates.

**Figure 4:**
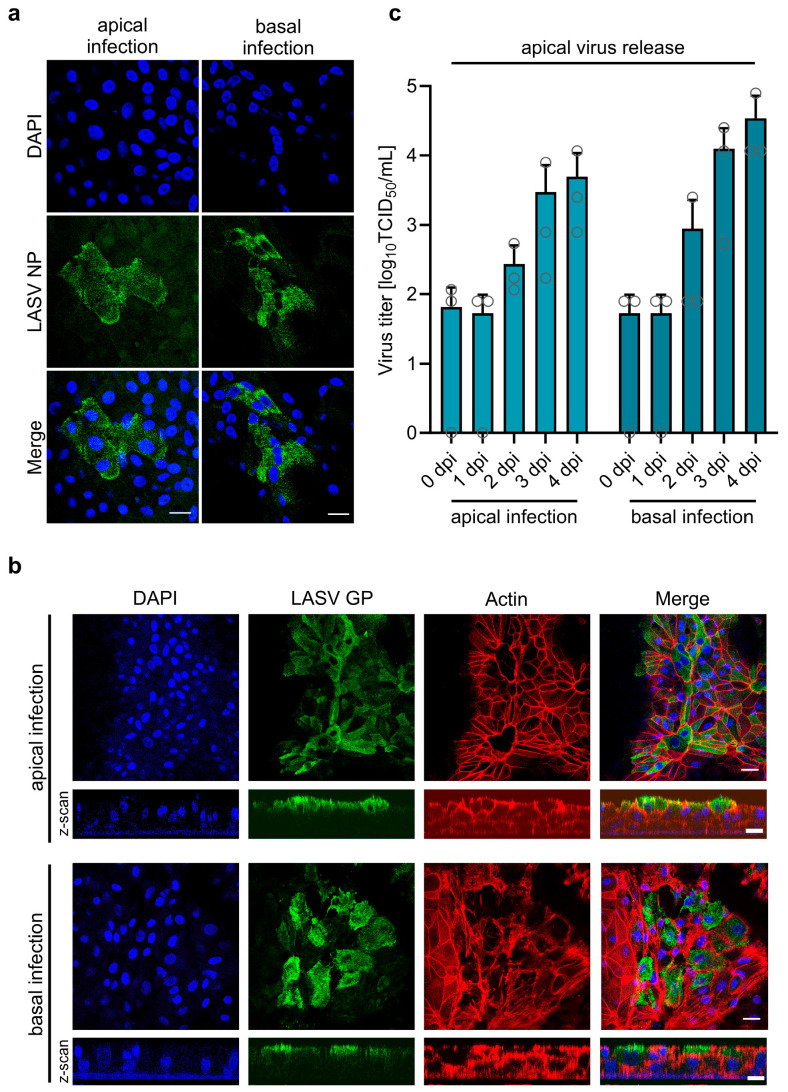
LASV entry and egress in primary human airway epithelial cells. (**a**,**b**) Differentiated HAECs cultured on Transwell inserts under ALI conditions were infected with 2.4 × 10⁴ PFU of LASV via either the apical or basolateral membrane. Infected cells were fixed with 4% PFA either at 48 hpi, and virus-positive cells were detected by immunostaining with a rabbit anti-LASV NP antibody (**a**), or at 4 dpi, where virus-positive cells were detected using a human anti-LASV GP antibody (**b**). Actin filaments were visualized with Phalloidin-TRITC staining, and cell nuclei were stained with DAPI. Scale bars, 20 µm (**a**) and 25 µm (**b**). (**c**) HAECs were infected with 8.75 × 10⁴ PFU of LASV via the apical or basolateral route. Virus titers in the apical medium were determined at the indicated time points post-infection using TCID_50_ analysis. Error bars represent the SEM of three biological replicates.

**Figure 5:**
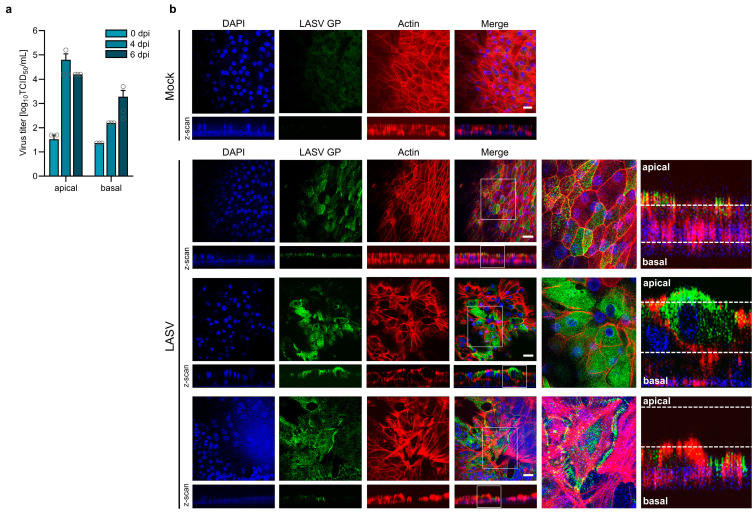
LASV infection induces actin cytoskeleton rearrangement. (**a**) Differentiated HAECs were infected with 1.2 × 10⁵ PFU of LASV simultaneously via both the apical and basolateral routes. Virus release was determined by TCID_50_ analysis at the indicated time points post-infection. Error bars represent the SEM of three biological replicates. (**b**) HAECs were infected with 2.4 × 10⁴ PFU of LASV via the apical side. Infected cells and mock-infected cells were fixed with 4% PFA at 4 dpi. Virus-positive cells were detected by immunostaining using a human monoclonal anti-LASV GP antibody. Actin filaments were visualized by Phalloidin-TRITC staining, and cell nuclei were stained with DAPI. Enlarged regions from the top-view images and z-scans are indicated by white frames. Scale bars, 25 µm.

**Figure 6:**
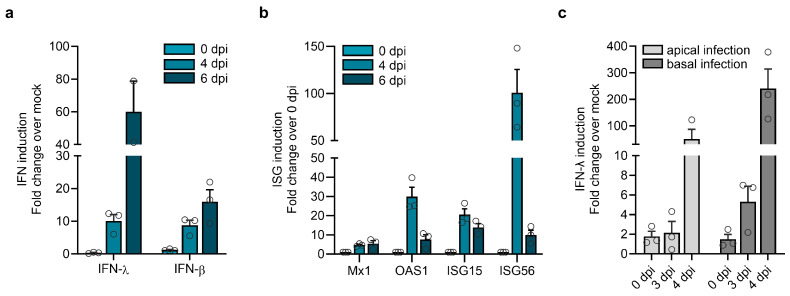
IFN induction by LASV infection. Differentiated HAECs were infected with 1.2 × 10^5^ PFU of LASV simultaneously via the apical and basolateral routes. Induction of IFN (**a**) and ISGs (**b**) was analyzed by quantitative RT-PCR. Error bars represent the SEM of three biological replicates. (**c**) Differentiated HAECs were infected with 1.2 × 10^5^ PFU of LASV via the apical or the basolateral route, and IFN-λ induction was determined by quantitative RT-PCR. Error bars represent the SEM of three biological replicates.

**Figure 7:**
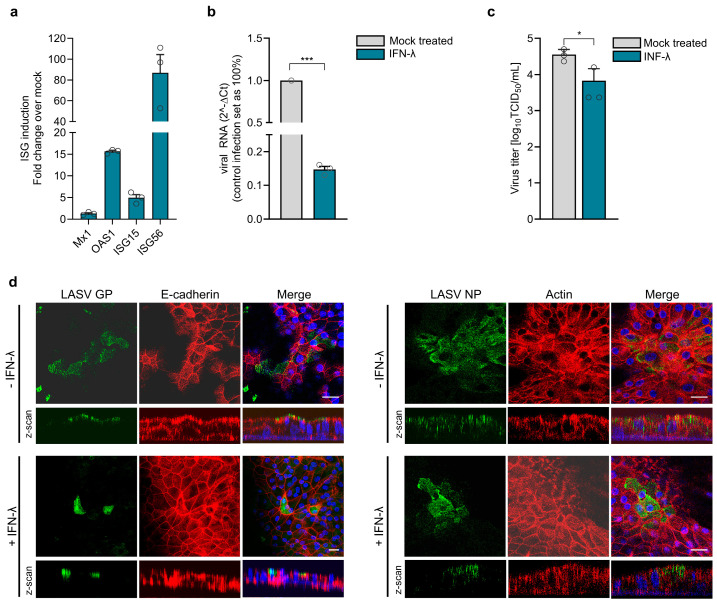
IFN induction and IFN-mediated antiviral responses during LASV infection. (**a**) Differentiated HAECs were stimulated for 16 h with human IFN-λ2, and ISG induction was analyzed by quantitative RT-PCR. (**b**–**d**) IFN-λ preincubated cells were infected with 1.2 × 10^5^ PFU of LASV simultaneously via the apical and basolateral routes. (**b**) At 48 hpi, viral replication was assessed by quantitative RT-PCR using viral RNA isolated from cell lysates. (**c**) Virus titers in the apical medium were determined by TCID_50_ analysis. Error bars represent the SEM of three biological replicates. Statistical significance was calculated using an unpaired *t*-test (* *p* = 0.032; *** *p* < 0.01). (**d**) Infected cells were fixed with 4% PFA at 48 hpi. Virus-positive cells were detected by immunostaining with anti-LASV NP or anti-LASV GP antibodies. Cell boundaries were visualized by actin or E-cadherin staining, and nuclei were stained with DAPI. Scale bars, 25 μm.

Primary human airway epithelial cells (HAECs) were obtained from Provitro (Berlin, Germany; primary human bronchial epithelial cells, HBEpCs) or LONZA (Basel, Switzerland; primary normal human bronchial epithelial cells, NHBE). HBEpCs were processed as previously described [43], and NHBEs were processed according to the manufacturer’s instructions. In brief, HAECs were first expanded in T75 flasks (Corning, Glendale, AZ, USA) in airway epithelial cell growth medium (AEGM; PromoCell, Heidelberg, Germany), supplemented with AEGM SupplementMix (PromoCell), or in bronchial epithelial cell growth basal medium (BEBM, LONZA), supplemented with SingleQuots^TM^ Supplements (LONZA), respectively. At 70% confluency, HAECs were detached, and 75,000 cells and 150,000 cells, respectively, were seeded on 12 mm Transwell culture inserts (0.4 μm pore size; Corning, Corning, NY, USA) coated with 0.01% rat-tail collagen type I (Sigma-Aldrich, Munich, Germany). After two days, the apical medium was removed, and the basal medium was replaced with a 1:1 mixture of supplemented AEGM:DMEM (Gibco) or BEBM:DMEM containing 50 ng/mL retinoic acid (ALI growth medium). The culture medium was changed every 2–3 days, and cells were cultured under ALI conditions for approximately 30 days. For weekly mucus extraction, primary cells were washed apically with phosphate-buffered saline (PBS). The apical washes were pooled, and mucins were concentrated by centrifugation. For monitoring cell polarization and barrier formation during the proliferation phase, the transepithelial electrical resistance (TEER) was measured. The development of a pseudostratified cell layer was assessed by histological examination, and differentiation into specific cell types (ciliated, basal, goblet, or club cells) was monitored by immunostaining. We performed a comparative characterization of HBEpC- and NHBE-derived ALI cultures in terms of cell-type composition, differentiation status, and barrier function, revealing no significant phenotypic differences. The characterization of the polarized and differentiated phenotype is exemplified using HBEpCs. HBEpCs were used in the experiments presented in Figure 1, Figure 2, Figure 3, Figure 4c and Figure S1, whereas NHBEs were used in the experiments shown in Figure 4a,b, Figure 5, Figure 6 and Figure 7.

### 2.3. Transepithelial Electrical Resistance

TEER measurements were performed with a volt–ohm meter (EVOM2; World Precision Instruments, Friedberg, Germany) three times a week. The apical and basolateral chambers were filled with ALI medium, and triplicate wells were taken for each measurement. In parallel, the resistance of an empty filter was determined and subtracted; the difference was multiplied by the growth area of 1.12 cm^2^. TEER values are expressed in Ω·cm^2^. Cells were used for experiments when the transepithelial electrical resistance of > 800 Ω·cm^2^ was reached.

### 2.4. Histology

Morphological features of differentiated HAEC were assessed by histological examination. The cell culture insert was removed from the Transwell filter holder, fixed with 4% PFA for 20 min at RT, and incubated in 70% ethanol for 15 min at RT. The filter was cut into 3–5 mm strips and embedded in paraffin. Sections of 1–5 µm thickness were prepared using a microtome (Leica, Wetzlar, Germany), deparaffinized, and stained with hematoxylin and eosin. Specimens were covered with mounting medium and were microscopically evaluated under the light microscope DMi1 (Leica, Wetzlar, Germany).

### 2.5. Antibodies

The following primary antibodies were used to detect specific cell types. For ciliated cells, a monoclonal mouse anti-β-tubulin Cy3-conjugated antibody (Sigma-Aldrich, Deisenhofen, Germany) was used. Club cells were identified with a polyclonal rabbit anti-CC16 antibody (BioVendor, Brno, Czech Republic), and basal cells with either a monoclonal rabbit anti-p63 antibody (Abcam, Cambridge, UK) or a monoclonal mouse anti-CK17 antibody (BD Biosciences, San Jose, CA, USA). Goblet cells were detected using either a monoclonal mouse anti-TFF3 antibody (nanoTools, Teningen, Germany) or a monoclonal mouse anti-MUC5Ac antibody (Acris, Rockville, MD, USA). Fibroblasts were stained with a monoclonal mouse anti-fibroblast antibody (clone TE-7; Merck, Darmstadt, Germany).

Mucus proteins were detected using a monoclonal mouse anti-MUC4 antibody and a rabbit anti-MUC16 antibody (both from Abcam). To stain cellular components, the following primary antibodies were used. Tight junctions (TJs) were visualized with a polyclonal rabbit antibody against zonula occludens (ZO-3) (Merck Millipore, Burlington, MA, USA), and adherens junctions (AJs) were labeled with a monoclonal mouse anti-E-cadherin antibody (BD Biosciences). Cytokeratins were detected with a monoclonal mouse anti-pan-keratin antibody (Merck Millipore), a monoclonal mouse anti-CK7 antibody (Agilent, Santa Clara, CA, USA), and a monoclonal mouse anti-CK8 antibody (BD Biosciences). The actin cytoskeleton was stained using TRITC-conjugated phalloidin (Sigma-Aldrich, Taufkirchen, Germany).

The LASV nucleoprotein (NP) was detected with a polyclonal rabbit antibody (Biozol, Eching, Germany), while LASV glycoprotein (GP) was stained with a monoclonal human antibody (37.7H, Absolute Antibody, Oxford, UK). VSVΔG/LASVGP was identified using a polyclonal rabbit serum against VSV (kindly provided by Wolfgang Garten, Institute of Virology, Marburg, Germany). H1N1 IAV was detected with a monoclonal mouse anti-NP antibody (kindly provided by Alexander Klimov, Centers for Disease Control and Prevention, Atlanta, GA, USA). Tubulin was stained with a monoclonal mouse anti-tubulin antibody (Sigma-Aldrich, Taufkirchen, Germany), and β-actin was labeled with a monoclonal mouse anti-β-actin antibody (Abcam).

For western blot analysis, secondary antibodies conjugated with Alexa Fluor 680 (anti-rabbit) and Alexa Fluor Plus 800 (anti-mouse) were used (Invitrogen, Rockford, IL, USA). For immunofluorescence staining, an anti-human secondary antibody labeled with FITC (DAKO, Glostrup, Denmark), as well as anti-rabbit and anti-mouse secondary antibodies labeled with Alexa Fluor 568 or 488 (Invitrogen, Rockford, IL, USA), were applied. In the mucus neutralization assay, HRP-labeled anti-rabbit and anti-mouse secondary antibodies (Agilent, Santa Clara, CA, USA) were used.

### 2.6. Western Blot Analysis

For the detection of specific airway cell marker proteins, differentiated HAEC were lysed in 4× Laemmli buffer. For the detection of mucus proteins, the apical washes were prepared as described above. Proteins were separated by SDS-PAGE and transferred to nitrocellulose membranes (GE Healthcare Life Science, Chicago, IL, USA) for western blot analysis. The Odyssey Infrared Imaging System (LI-COR Biosciences, Lincoln, NE, USA) was used for visualization of the proteins. All gels/blots were derived from the same experiment and were processed in parallel. If several gels were needed to accommodate all samples, all samples originated from the same experiment and were processed in parallel.

### 2.7. Immunofluorescence Analysis

VeroE6 cells and HFF cells were seeded on coverslips, and HAECs were seeded on Transwell culture inserts and cultivated until the formation of a differentiated cell layer. For protein staining, the cells were fixed with 4% paraformaldehyde (PFA) for 20 min at RT, and incubated with 0.1 M glycine for 10 min. After permeabilization with 0.1% Triton X-100 for 10 min and blocking with 0.2% bovine serum albumin (BSA), the cells were incubated with specific primary antibodies diluted in BSA for 1.5 h at 4 °C. Secondary antibodies were added and incubated for 1.5 h at 4 °C. Cell nuclei were stained with 0.5 µg/mL 4′,6-diamidino-2-phenylindole (DAPI; Invitrogen, Rockford, IL, USA), and the cells were mounted with Fluoroshield (Sigma–Aldrich, Taufkirchen, Germany) for imaging. For staining of LASV proteins after infection, the cultures were incubated with primary antibodies diluted in 0.2% BSA for 1 h at RT, followed by incubation for 1 h at RT with secondary antibodies conjugated with Alexa Fluor 488 or 568 and supplemented with 0.5 µg/mL DAPI. The cells were then incubated for 48 h in 4% PFA and mounted in Fluoroshield. Images were captured on a confocal microscope (TCS SP5 II, Leica, Wetzlar, Germany). A 63× objective lens magnification was used, and image processing was performed using Fiji ImageJ Java 8 software.

### 2.8. mRNA Quantification by Quantitative Reverse-Transcriptase PCR

The RNeasy Mini Kit (Qiagen, Hilden, Germany) was used to isolate cellular mRNA, and the QiAmp viral RNA Mini Kit (Qiagen) was used to isolate viral RNA from supernatant according to the manufacturer’s instructions. cDNA was synthesized using the QuantiTect Reverse Transcription Kit (Qiagen), and RNA was quantified by real-time PCR using the SYBR Green PCR Master Mix (Fermentas, Vilnius, Lithuania) and PCR cycler (Step One; Applied Biosystems, Foster City, CA, USA). Measurements were performed in triplicate. Target genes included interferon (IFN)-α, IFN-β, IFN-λ, IFN-induced GTP-binding protein Mx1, 2′-5′-oligoadenylate synthetase 1 (OAS1), IFN-stimulated gene (ISG) 15, ISG56, and LASV GP. GAPDH was used as the housekeeping gene, and the relative gene expression was calculated by the 2^−ΔΔCt^ method using Step One 2.1 software (Applied Biosystems). The forward and reverse primer sequences will be provided upon request.

### 2.9. Apical and Basolateral Infection

For infection studies, differentiated HAECs were extensively washed to remove the mucus from the cell surface. Cells were then infected from the apical and basal sides with LASV using 1.2 × 10^5^ PFU for growth kinetics studies and 2.4 × 10^4^ PFU for immunofluorescence staining. After virus adsorption at 37 °C for 60 min, cells were washed three times with PBS and further cultivated under ALI conditions. Virus titers in the apical and basal supernatants were determined by the TCID_50_ method as described previously [39]. To determine the induction of ISGs in response to IFN stimulation, differentiated HAECs were stimulated from the basal side for 16 h with 10 ng/mL of recombinant human IFN-λ2 (IL-28A; PeproTech, Rocky Hill, NJ, USA). IFN-treated cells were washed and infected with LASV, as described above. At 48 h post-infection, cell lysates and cell culture supernatants were harvested for RNA isolation and TCID_50_ analysis, respectively.

### 2.10. Mucus Inhibition Assay

Concentrated airway mucus was serially diluted (from undiluted to 1:8192), and each dilution was incubated for 1 h at 37 °C with either 800 PFU of VSVΔG/LASVGP or 1400 focus-forming units (FFUs) of IAV. The virus–mucus mixtures were added to confluent VeroE6 cells (VSVΔG/LASVGP) and MDCK II cells (IAV), respectively, and incubated for 8 h at 37 °C to complete a single replication cycle. Then, cells were fixed with 4% PFA overnight and incubated with permeabilization buffer (0.1% Triton X-100, 100 mM glycine in PBS) for 15 min. Primary and secondary antibodies were diluted in PBS, containing 10% normal horse serum and 0.1% tween 20, incubated for 1 h at RT, and visualized by staining with TrueBlue (KPL, Gaithersburg, MD, USA). A rabbit anti-VSV antibody was used for VSVΔG/LASVGP-infected cells, and a mouse anti-NP antibody was used for H1N1-infected cells. HRP-conjugated secondary antibodies were used. Infected cells were counted (light microscope DMi1, Leica, Wetzlar, Germany), and the means of the triplicate samples were calculated. Infected cells without mucus treatment were used as the negative control and set as 100%. The number of cells infected in the presence of mucus was expressed as a percentages relative to the positive control.

## 3. Results

### 3.1. Characterization of HAEC Cultured Under ALI Conditions

Primary HAECs cultivated under ALI conditions can differentiate into a polarized cell layer that includes all major cell types found in the respiratory epithelium in vivo, such as ciliated, basal, and mucus-secreting cells [43,44]. Therefore, differentiated HAECs represent an important cell culture model to analyze viral replication in the human respiratory epithelium. Here, we evaluated HAECs for their susceptibility to LASV. To characterize their epithelial phenotype, we first examined the expression of cytokeratins (CKs), intermediate filaments typically found in epithelial cells [45]. As shown in Figure 1a, immunofluorescence analysis with a pan-CK antibody confirmed cytokeratin expression in all cells, exhibiting a characteristic cellular distribution [46]. Staining with the specific fibroblast cell marker TE-7 showed a clear positive signal in control cells (human foreskin fibroblasts, HFF), while no TE-7 expression was observed in HAECs (Figure 1b). To assess the ability to develop a polarized cell layer, HAECs were seeded on Transwell inserts and cultivated under ALI conditions for several weeks. The polarization was monitored by measuring TEER values. As shown in Figure 1c, TEER started to increase after 7 days, reaching a maximum of 878 Ω·cm^2^ after 28 days. As expected for highly polarized epithelial cells, the TJ marker ZO-3 and the AJ protein E-cadherin displayed the characteristic honeycomb-like staining pattern (Figure 1d).

### 3.2. HAECs Differentiate into a Pseudostratified Epithelium

To confirm that HAECs cultivated under ALI conditions developed a differentiated pseudostratified respiratory epithelial layer, we analyzed the expression of CKs, which serve as markers for the differentiation status of epithelial cells [47]. CK7 and CK8 are expressed by secretory-active epithelial cells of the respiratory tract [48], whereas CK17 functions as a marker for undifferentiated epithelial cells, such as basal cells [49]. Immunofluorescence analysis revealed the expression of all three CKs in HAECs, indicating that the epithelium comprises both differentiated and undifferentiated cells (Figure 2a). To characterize the cell types present in HAEC cultures, we used specific markers for ciliated cells (ß-tubulin), goblet cells (TFF-3), club cells (CC16), and basal cells (p63). As shown in Figure 2b, the most abundant cell type was ciliated epithelial cells. In addition, the multilayered epithelium included secretory goblet and club cells as well as non-differentiated basal cells. The presence of ciliated, club, and basal cells was also confirmed by western blot analysis. When we compared non-differentiated and differentiated cultures (day 0 and day 50), we found ß-tubulin (ciliated cells) and CC16 (club cells) in differentiated cells, while p63 (basal cells) was mostly expressed in non-differentiated HAECs (Figure 2c). The formation of a pseudostratified epithelium with an apical ciliary border was also confirmed in HE-stained histological samples (Figure 2d). Since mucins secreted by differentiated secretory cells (goblet and club cells) are another hallmark of differentiated HAEC cultures [50], we analyzed apical washes of the ALI cultures for the presence of MUC4 and MUC16 by western blot analysis. Tethered mucins were detected in cell-associated as well as in apical washes of the ALI cultures from day 16 onward (Figure 2e). Together, these results demonstrate that HAEC cultivated under ALI conditions differentiated into a functional, respiratory epithelium-like tissue, containing all typical cell types and secreting mucins, effectively mimicking airway epithelia.

### 3.3. Effect of Airway Mucus on LASV GP-Mediated Virus Entry

The binding of the virus to its specific receptor on the host cell plasma membrane is a crucial prerequisite for successful infection. One defense mechanism is the secretion of soluble receptor analogs that bind free viruses, thereby preventing their attachment to cell surface receptors. For example, sialic acids, which serve as entry receptors for influenza A viruses (IAVs), are secreted and concentrated in the mucus, where they can compete with IAV binding to airway epithelial cells and inhibit influenza infection [42]. Given that one major LASV receptor, α-DG, was also detected in human airway mucus [51], we wanted to investigate whether airway mucus interferes with LASV attachment to HAECs. For this reason, we harvested and concentrated the mucus secreted from the apical surface of differentiated HAEC cultures. As a control, we preincubated IAV H1N1 with different mucus dilutions prior to infecting MDCK II cells. Virus-infected cells were immunostained, and the relative reduction of the number of infected cells was determined. Figure 3a shows a dose-dependent neutralization of IAV, consistent with earlier findings [42]. To assess the inhibitory effect of airway mucus on LASV GP-mediated entry, we used a replication-competent vesicular stomatitis virus (VSV) expressing LASV GP (VSVΔG/LASVGP). This BSL2 surrogate system allowed us to quantify GP-mediated attachment and entry in the context of LASV neutralization [52]. VSVΔG/LASVGP was preincubated with mucus dilutions prior to infecting VeroE6 cells. Unlike IAV, VSVΔG/LASVGP entry was unaffected, regardless of the mucus concentration, suggesting that human airway mucus does not inhibit LASV GP-mediated viral entry (Figure 3b).

### 3.4. Entry and Release of LASV in Polarized Human Airway Epithelial Cells

Depending on the route of infection, viruses can enter the respiratory epithelium via the apical or basal side. In vivo, during initial infections when LASV enters a new host via the respiratory route, airway epithelia are infected from the apical side. At later stages of systemic infection, LASV circulating in the blood must access epithelial cells from the basolateral side. To determine from which side LASV can initiate infection in polarized, differentiated HAECs, we infected the cells by adding LASV to either the apical or basal filter chamber and monitored the infection at 48 h post-infection (hpi) by LASV NP-specific immunostaining. As shown in Figure 4a, LASV-positive foci were detected in both types of infected cultures, indicating that LASV can infect the airway epithelium from both the apical and basolateral sides.

Directed virus release via the apical or basolateral side is associated with a polarized distribution of viral envelope proteins [53,54]. We previously demonstrated that LASV GP localizes to the apical plasma membrane in polarized kidney epithelial cells, consistent with predominant virus release from the apical surface [35]. To determine whether apical transport is a common mechanism of LASV GP in polarized epithelial cells, we analyzed its cellular localization in differentiated HAECs. To investigate this, HAECs were infected with LASV from either the apical or basolateral side. Confocal microscopy analysis revealed a strict apical localization of LASV GP, regardless of the route of infection (Figure 4b). To further assess whether the apical localization of LASV GP corresponds to apical virus release, we infected HAECs via the apical or basolateral side and measured LASV titers in the apical chamber. As shown in Figure 4c, LASV was efficiently released into the apical compartment, independent of the infection route. Furthermore, the data demonstrate productive multi-cycle replication in differentiated HAECs, with viral titers increasing over time. Similar results have been observed in human intestinal epithelial Caco-2 cells infected with Lymphocytic choriomeningitis mammarenavirus (LCMV) or Mopeia mammarenavirus (MOPV), where virus particles were released from the apical surface following either apical or basolateral infection [55]. Our data suggest that apical virus shedding from infected respiratory epithelial cells may facilitate LASV transmission through airway secretions.

### 3.5. Basolateral Release of LASV at Late Stages of Infection Is Associated with Actin Cytoskeleton Rearrangement

Basolateral virus release from infected respiratory epithelial cells may play a critical role in LASV spread into underlying tissues, contributing to subsequent systemic dissemination. To determine whether HAECs support basolateral LASV release, we compared virus titers in the apical and basolateral media. As shown in Figure 5a, progeny virus particles were predominantly released from the apical surface.

At 4 dpi, apical virus titers had increased 1000-fold compared to the inoculum (0 dpi), whereas infectious LASV titers in the basal medium had only increased tenfold. However, the observed two-log difference between apical and basal viral titers at day 4 was not maintained over time; instead, it gradually decreased. By day 6 post-infection, this difference was reduced to one log. While apical viral titers declined at day 6, the amount of infectious virus in the basal chamber increased. These findings suggest that LASV primarily egresses via the apical membrane, but its polarized release is partially lost at later stages of infection. Since the actin cytoskeleton plays a crucial role in forming and maintaining the epithelial barrier and is therefore essential for epithelial integrity [56], we examined the effects of LASV infection on actin filaments at day 4 post-infection, when limited basolateral virus titers were observed. Confocal microscopy revealed distinct stages of infection, each associated with specific alterations in actin organization (Figure 5b). In regions corresponding to the initial stage of infection, cells exhibited regular actin staining and a strictly apical localization of LASV GP (Figure 5b, LASV, upper panel). In contrast, regions indicative of later stages of infection exhibited a reorganization of actin filaments (Figure 5b, LASV, middle and lower panels), characterized by disruptions in the typical honeycomb-like pattern at cell boundaries, as compared to the actin staining in the mock control. Notably, the LASV GP signal, initially restricted to the apical surface, was also detected in the middle regions of the pseudostratified epithelium, beneath the apical membrane (Figure 5b, LASV, middle panel). In advanced stages, where the actin filament network was no longer detectable, LASV GP was distributed across the entire plasma membrane of infected cells (Figure 5b, LASV, lower panel), indicating a loss of epithelial polarity. These findings indicate that LASV infection can induce structural disorganization of the actin cytoskeleton, potentially compromising epithelial integrity. The resulting loss of polarity may facilitate LASV spread to the basal side of the respiratory epithelium, explaining the increase in basal virus titers observed at 6 dpi.

### 3.6. Type I and III Interferon Induction in HAECs in Response to LASV Infection

In addition to type I IFNs (IFN-α/β), respiratory epithelial cells can secrete and respond to type III IFNs (IFN-λ). To determine the role of IFNs in LASV replication, we infected differentiated HAECs with LASV and measured the IFN induction. As shown in Figure 6a, we observed a predominant type III IFN response in comparison to type I IFN. Similar findings have been reported for Nipah virus-infected primary airway epithelial cells [57]. Infection of HAECs also led to the upregulation of several specific ISGs, which act downstream of both type I and III IFN pathways and have been implicated in responses to LASV [58,59] (Figure 6b).

To assess whether donor variations might influence the induction and magnitude of type I and III IFN responses, we analyzed the IFN response to LASV infection in differentiated HAEC derived from two additional donors. Consistent with the results described before, both donors exhibited a robust type III IFN response and upregulation of ISGs but showed limited IFN-β responses (Figure S1). These findings suggest that differentiated airway epithelial cells from individual donors share the ability to respond to LASV infection by efficient IFN-λ induction. Since LASV can infect bronchial epithelial cells via both the apical and basolateral sides, we wanted to determine whether the route of infection influences IFN-λ induction. Therefore, we compared IFN-λ induction following apical or basolateral LASV entry. While IFN-λ mRNA expression in differentiated HAECs was induced after LASV infection from both sides, infection via the basolateral side exhibited a more pronounced effect (Figure 6c). This suggests that RNA sensing and IFN induction are enhanced when LASV enters HAECs via the basolateral surface.

### 3.7. Pretreatment of HAECs with Type III Interferon Restricts LASV Replication

As IFN-λ is the major antiviral IFN induced during LASV infection of airway epithelia, we wanted to determine if pretreatment with IFN-λ can block LASV replication. Therefore, differentiated HAECs were pretreated with recombinant IFN-λ2 (IL-28A) for 16 h prior to infection. Exogenous IFN treatment led to the upregulation of ISGs, confirming that HAECs respond to IFN-λ and can establish an antiviral state (Figure 7a). Compared to untreated cells, IFN-λ-treated HAECs showed significantly reduced viral RNA levels at 48 h post-infection (Figure 7b). Consistently, viral titers were also lower in IFN-λ-treated cells (Figure 7c). To assess whether IFN-λ pretreatment affects the apical distribution of LASV GP or reduces the number of infected cells, we infected IFN-λ-stimulated and untreated HAECs via the apical and basolateral membranes and immunostained the cells for LASV GP and NP proteins at 48 hpi. As shown in Figure 7d, IFN-λ pretreatment did not alter GP distribution. However, staining for GP and NP revealed that IFN-λ pretreatment reduced the number of infected cells. This suggests that the observed reduction in virus release is not due to impaired GP transport but rather to a general downregulation of viral replication and spread.

## 4. Discussion

In this study, we demonstrate that differentiated primary HAECs grown under ALI conditions are permissive to LASV infection and support virus replication, enabling viral entry into the respiratory epithelium through both the apical and basolateral membranes. In contrast, LCMV exhibits restricted entry via the basolateral membrane in primary airway epithelial cells. Interestingly, the receptor α-DG is present on both apical and basolateral surfaces in these cells, making the strict basolateral entry of LCMV difficult to explain [60]. The bipolar entry of LASV observed in our study is consistent with the unrestricted expression of α-DG. However, it cannot be excluded that additional LASV receptors contribute to apical viral entry. Future studies are needed to identify the specific receptors on the apical and basolateral membranes that facilitate LASV infection of polarized airway epithelial cells from both sides. In contrast to bipolar virus entry, newly synthesized virus particles were predominantly released from the apical cell surface. LASV GP plays a key role in facilitating efficient virus replication and spread. In the intact epithelium, GP is strictly localized to the apical membrane, potentially guided by the numerous N-glycans on GP, which can serve as apical sorting signals [61,62]. Enhanced LASV release has also been observed through the apical membrane in polarized MDCK cells [35]. These findings align with previous studies on other mammarenaviruses, such as New World Junín and Tacaribe viruses, which also preferentially exhibit apical release from polarized epithelial cells [63]. Infection of polarized human intestinal epithelial Caco-2 cells with LCMV or MOPV revealed that virus particles were primarily released from the apical side after apical infection, while basolateral infection led to a similar level of virus release from both the apical and basolateral surfaces [55]. In contrast, LCMV release from infected polarized primary human airway epithelial cultures occurred exclusively via the basolateral cell surface [60]. These observations emphasize the impact of cell type-specific factors on viral egress patterns. Further research is needed to identify the molecular determinants underlying these variations in viral release.

Previous studies have demonstrated the presence of LASV RNA in saliva and throat swab samples from Lassa fever patients, as well as both viral RNA and infectious LASV in mucosal specimens obtained from animal models [64,65,66]. Although the precise cellular origin of the detected viral RNA and infectious LASV remains unclear and may involve various tissues or immune cells, our findings suggest that respiratory epithelial cells can contribute to viral shedding and may thus play a role in human-to-human transmission via respiratory secretions. These observations underscore the need for further investigation into the role of the respiratory epithelium in LASV transmission.

We observed that basolateral virus release is limited and delayed compared to apical release. Confocal microscopy analysis revealed basolateral localization of GP at advanced stages of infection, when cell polarity was partially lost, impairing polarized protein transport. The LASV-induced loss of cell polarity and epithelial integrity in human airway epithelial cells may have multiple molecular causes. Viral infection could directly or indirectly affect polarity complexes, regulatory proteins, or components of the host cell cytoskeleton. At advanced stages of LASV infection, we observed a rearrangement of the actin cytoskeleton in differentiated HAECs, potentially leading to the disruption of epithelial barrier integrity. Such a loss of cell integrity may facilitate LASV transmigration to the basal side after spreading through the epithelial cell layer, potentially representing a pathogenic mechanism by which LASV overcomes the protective airway epithelial barrier, leading to systemic spread. Similarly, during Zika virus infection, the interaction between the viral envelope E protein and F-actin has been shown to reorganize the actin network in later stages, thereby compromising the integrity of the blood–testis barrier [67]. Dengue virus infection has also been reported to induce actin cytoskeleton reorganization, which is thought to contribute to endothelial barrier dysfunction and increased vascular permeability observed in dengue hemorrhagic fever [68,69]. Moreover, actin cytoskeleton disruption is a common strategy used by various neurotropic viruses to compromise the blood–brain barrier [70]. Future studies should elucidate the precise mechanisms through which LASV interferes with the actin network and how this affects epithelial barrier function and basolateral virus release.

Interference with TJ and AJ proteins is an alternative pathogenic mechanism employed by several viruses to compromise the epithelial barrier and access specific receptors on the basolateral membrane [71,72,73]. LASV efficiently infects the polarized respiratory epithelium via the apical membrane, suggesting that epithelial barrier disruption is not required for initial infection. However, modulation of junctional complexes may play a role in how LASV overcomes the airway epithelium. Previous studies have shown that LASV infection does not disrupt the integrity of TJ and AJ in polarized kidney epithelial cells [35]. We observed cytopathic effects at advanced stages of infection in HAECs. However, the titers of apically and basally released viruses remained unequal even six days post-infection, suggesting that LASV infection does not compromise the overall integrity of the epithelial barrier. The impairment of epithelial integrity during LASV infection may be driven by both viral and cellular factors. For example, studies on respiratory syncytial virus (RSV) and rhinoviruses have shown that viral infections can disrupt components of the TJ and AJ complexes [74,75]. In the case of RSV, while TJ and AJ protein expression levels remained unchanged, the loss of polarity was associated with virus-induced disassembly of these junctional proteins [76]. The adenovirus protein E4-ORF1 directly binds to and inactivates the TJ scaffold proteins MUPP1, MAGI-1, and ZO-2 [77]. Impaired TJ function increases intercellular permeability, facilitating the transmigration of progeny virions from infected respiratory epithelial cells to the basolateral side [78]. The Dengue virus NS1 protein interacts with MMP-9, leading to the degradation of the adhesion molecule β-catenin and the TJ proteins ZO-1 and ZO-2. Impaired endothelial cell adhesion and TJ function result in endothelial hyperpermeability and vascular leakage [79]. LASV GP has been shown to interact with several AJ and TJ proteins, though the mechanistic consequences of these interactions remain unclear [80]. Studies on chronic respiratory diseases have shown that IFNs, particularly IFN-λ, and TNF-α influence the regulation and integrity of the epithelial barrier [81,82]. Furthermore, IFN-λ has been shown to cause airway epithelial barrier damage in response to synthetic viral RNA [83]. Since IFN-λ is effectively induced in HAECs during LASV infection, it may also play a role in impaired barrier function. However, IFN-λ treatment of HAECs did not result in changes in the cellular distribution of actin and E-cadherin, leaving the role of IFN-λ in epithelial barrier disruption under our experimental conditions unclear. Additional studies are needed to better define the impact of LASV-induced IFN-λ on epithelial barrier integrity.

Type I and type III IFNs share similarities in their induction and antiviral activity but differ in receptor expression patterns, ISG expression, and negative regulation of IFN signaling (reviewed in [84]). Previous studies have shown that LASV nucleoprotein NP and the matrix protein Z inhibit the induction of type I IFNs [85,86]. In our study, we observed a robust induction of IFN-λ, but not IFN-α/β, in LASV-infected HAECs, suggesting that type I and type III IFN induction differ in their susceptibility to antagonism by LASV. Huang et al. reported that the vaccinia virus protein B18 acts as a specific antagonist of type I IFN, but not type III IFN, by blocking STAT1 activation [87]. For LASV, it is known that NP antagonizes IRF3, while the Z protein inhibits RIG-I-like receptor signaling [85,86,88]. IFN-λ is the key cytokine in the immune response to respiratory infections. IFN-λ-specific receptors are highly expressed in epithelial cells, including HAECs [57,89]. The expression of INF-λ can be induced by viral dsRNA, which is mainly sensed by cytosolic RIG-I or endosomal TLR3 [90,91]. We observed that LASV induces IFN-λ expression following both apical and basal infection. However, IFN-λ induction was notably higher when LASV entered HAECs from the basolateral side. This raises the question of whether this difference is due to faster viral uptake or more efficient sensing by TLR3 for viruses entering basolaterally. Guillot et al. showed that TLR3 is exclusively expressed intracellularly in airway epithelial cells, whereas Ioannidis et al. found that TLR3 is present in the cytoplasm as well as on both the apical and basolateral cell surfaces [92,93]. In polarized intestinal epithelial cells, TLR3 was localized basolaterally, leading to a stronger induction of IFN-λ upon basolateral virus infection [94]. An asymmetric distribution of TLR3 could also explain the increased IFN-λ induction observed when HAECs were infected with LASV from the basolateral side. Future studies are needed to fully characterize the cellular distribution of TLR3 in differentiated HAECs. Alternatively, the observed differences in response may stem from variations in infection efficiency or differences in the specific cell types targeted by LASV during apical versus basolateral entry. LASV replication persisted in HAEC despite a robust type III IFN and ISG response, as demonstrated by the continuous increase in virus titers over time. Our findings align with previous in vivo studies showing that IFN-λ does not effectively restrict LASV replication [95]. However, prestimulation of HAECs with IFN-λ reduced viral replication and virus release, highlighting its antiviral activity against LASV. This suggests that IFN-λ administration could serve as a potential prophylactic or early therapeutic strategy for LASV infection in the respiratory tract.

In conclusion, we demonstrate that human bronchial epithelial cells can be infected by LASV from both the apical and basolateral surfaces. This finding suggests that airway epithelial cells may contribute to initial viral replication during entry via the respiratory tract. Moreover, they may facilitate viral shedding through airway secretions during systemic infection, when LASV reaches the respiratory mucosa from the basal side—a hypothesis further supported by the efficient apical release of progeny virus particles. Collectively, these results indicate that the human respiratory tract is not only a critical entry point for LASV but may also play a role in its transmission and dissemination.

## Data Availability

The data presented in this study are available on request from the corresponding author.

## Supplementary Materials

The following supporting information can be downloaded at: https://www.mdpi.com/article/10.3390/v17050592/s1, Figure S1: Donor variability in IFN and ISG induction.
