# Supplementary material for: Lassa Virus Infection of Primary Human Airway Epithelial Cells
Source: Viruses. 2025 Apr 22;17(5):592. doi: 10.3390/v17050592 (PMC12116150; doi:10.3390/v17050592)
Supplement: Supplementary file 1 [file viruses-17-00592-s001.zip › viruses-3549101-supplementary.pdf]

Supplementary Information

Title of manuscript: Lassa Virus Infection of Primary Human Airway Epithelial Cells

Author list: Helena Müller-Kräuter, Sarah Katharina Fehling, Lucie Sauerhering, Birthe Ehlert, Janine Koepke, Juliane Schilling, Mikhail Matrosovich, Andrea Maisner and Thomas Strecker

Supplementary Figure S1

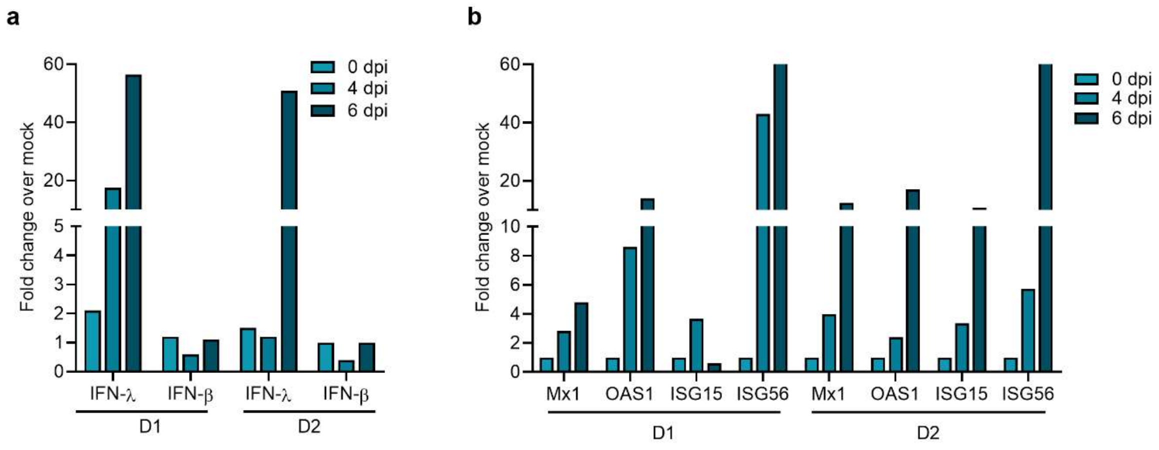

Figure S1. Donor variability in IFN and ISG induction. Differentiated HAEC of two different donors (D1 and D2) were infected with 1.3 ×10^5 PFU of LASV simultaneously via both the apical and basolateral routes. The induction of IFN **(a)** and ISGs **(b)** was assessed by quantitative RT-PCR.
